# The Effect of Disease Modifying Therapies on Brain Atrophy in Patients with Relapsing-Remitting Multiple Sclerosis: A Systematic Review and Meta-Analysis

**DOI:** 10.1371/journal.pone.0116511

**Published:** 2015-03-10

**Authors:** Georgios Tsivgoulis, Aristeidis H. Katsanos, Nikolaos Grigoriadis, Georgios M. Hadjigeorgiou, Ioannis Heliopoulos, Constantinos Kilidireas, Konstantinos Voumvourakis

**Affiliations:** 1 Second Department of Neurology, “Attikon” Hospital, School of Medicine, University of Athens, Athens, Greece; 2 Department of Neurology, The University of Tennessee Health Science Center, Memphis, Tennessee, United States of America; 3 International Clinical Research Center, Department of Neurology, St. Anne’s University Hospital in Brno, Brno, Czech Republic; 4 Department of Neurology, School of Medicine, University of Ioannina, Ioannina, Greece; 5 Second Department of Neurology, “AHEPA” University Hospital, Aristotelion University of Thessaloniki, Thessaloniki, Macedonia, Greece; 6 Department of Neurology, University Hospital of Larissa, University of Thessaly, Larissa, Greece; 7 Department of Neurology, Alexandroupolis University Hospital, Democritus University of Thrace, Alexandroupolis, Greece; 8 First Department of Neurology, “Eginition” Hospital, School of Medicine, University of Athens, Athens, Greece; University of Oxford, UNITED KINGDOM

## Abstract

**Background:**

The aim of the present meta-analysis was to evaluate the effect of disease-modifying drugs (DMD) on brain atrophy in patients with relapsing-remitting multiple sclerosis (RRMS) using available randomized-controlled trial (RCT) data.

**Methods:**

We conducted a systematic review and meta-analysis according to PRISMA guidelines of all available RCTs of patients with RRMS that reported data on brain volume measurements during the study period.

**Results:**

We identified 4 eligible studies, including a total of 1819 RRMS patients (71% women, mean age 36.5 years, mean baseline EDSS-score: 2.4). The mean percentage change in brain volume was found to be significantly lower in DMD versus placebo subgroup (standardized mean difference: -0.19; 95%CI: -0.27–-0.11; p<0.001). We detected no evidence of heterogeneity between estimates (I^2^ = 30%, p = 0.19) nor publication bias in the Funnel plots. Sensitivity analyses stratifying studies according to brain atrophy neuroimaging protocol disclosed no evidence of heterogeneity (p = 0.16). In meta-regression analyses, the percentage change in brain volume was found to be inversely related with duration of observation period in both DMD (meta-regression slope = -0.03; 95% CI: -0.04–-0.02; p<0.001) and placebo subgroups (meta-regression slope = -0.05; 95% CI: -0.06–-0.04; p<0.001). However, the rate of percentage brain volume loss over time was greater in placebo than in DMD subgroup (p = 0.017, ANCOVA).

**Conclusions:**

DMD appear to be effective in attenuating brain atrophy in comparison to placebo and their benefit in delaying the rate of brain volume loss increases linearly with longer treatment duration.

## Introduction

Longitudinal studies have shown that brain atrophy is a significant predictor of subsequent long-term neurologic deterioration, impaired life quality and sustained disability in patients with multiple sclerosis (MS) [1]. Brain atrophy was also found to be related to cognitive deficits (even in the early stages of the disease) mood disturbances, sexual dysfunction and personality disorders [1–3].

Brain atrophy may occur in early disease stages, progresses more rapidly than in healthy individuals and appears to advance relentlessly throughout the course of MS, independent of the underlying disease subtype when adjusted for baseline volume [4,5]. A recent meta-analysis underlined that the treatment effect of available immunomodulatory therapies on brain atrophy was related to the effect on disability progression in patients with relapsing-remitting MS (RRMS). Interestingly, the former association was independent of the treatment effect on active lesions in magnetic resonance imaging (MRI) [6]. Prospective cohort studies and randomized controlled trials (RCTs) have reported variable results regarding the potential protective effects of different disease modifying drugs (DMD) on brain atrophy [7–10, References 1–13 in S1 File].

To the best of our knowledge the efficacy of DMD in attenuating brain atrophy in MS patients has not been investigated systematically using a meta-analytical approach. In view of the former considerations we conducted a systematic review and meta-analysis to evaluate the effect of available DMD on brain atrophy in patients with RRMS using available RCT data.

## Methods

### Trial identification and data abstraction

This meta-analysis has adopted the Preferred Reporting Items for Systematic Reviews and Meta-Analyses (PRISMA) guidelines for systematic reviews and meta-analyses [11]. Eligible placebo-control randomized clinical trials (RCTs) of patients with RRMS that reported changes in brain volume during the study period were identified by searching MEDLINE, SCOPUS and the CENTRAL Register of Controlled Trials. The combination of search strings that was used in all database searches included the terms: “relapsing-remitting multiple sclerosis”, “RRMS”, “brain atrophy” and “brain volume”. The complete search algorithm that was used in MEDLINE search is available in the S1 File. No language or other restrictions were imposed. Last literature search was conducted on August 23th, 2014. Reference lists of all articles that met the criteria and of relevant review articles were examined to identify studies that may have been missed by the database search.

All retrieved studies were scanned independently by two reviewers (GT & AHK) to include only placebo-control RCTs of RRMS patients that reported changes in brain volume during the study period. We excluded from the final analysis: 1. Observational studies, 2. case series, 3. case reports, 4. RCTs without placebo subgroups, 5. studies reporting the use of drugs that are still not officially approved (e.g. laquinimod, cladribine), and 6. studies reporting brain volume data with median values or not providing measures of dispersion in the form of standard deviation (SD). We considered that including trials reporting brain volume loss both in medians (with corresponding confidence intervals) and in means (with corresponding SDs would constitute another source of heterogeneity in our meta-analysis that could probably lead to incorrect pooled outcomes, given the differences in dispersion measures between comparison groups in the other studies. Thus we decided in our study protocol and analysis plan that was formulated according to PRISMA guidelines [11] and Cochrane Handbook for Systematic Reviews of Interventions [12] to exclude per se studies that did not provide the corresponding SDs for both treatment and placebo groups. In case of disagreement regarding the literature search results between the two coauthors, the senior coauthor was consulted (KV) and disagreement was resolved with consensus.

In each study that met the inclusion criteria for the quantitative analysis a predefined 7-point quality control was used to address for biases. For each quality item the corresponding risk of bias was categorized as low, high or unclear according to the suggestions by Higgins et al [12]. Quality control and bias identification was performed by three independent reviewers (GT, AHK, KV) and all emerging conflicts were resolved with consensus.

Data on brain volume changes in all subgroups between time points, or during time intervals within studies were extracted independently by the two authors, who performed the literature search (GT, AHK).

### Statistical analyses

Unadjusted mean differences of percentage changes in brain volumes between treatment and placebo subgroups were pooled as standardized mean differences (SMDs). SMD estimates were calculated as the mean differences divided by the corresponding pooled standard deviations and were subsequently interpreted using a general rule of thumb reported by Cohen, in which an SMD of 0.2 represents a small effect, an SMD of 0.5 represents a medium effect, and an SMD of 0.8 or larger represents a large effect [13]. A random-effects model (DerSimonian Laird) was used to calculate the pooled SMDs.

Heterogeneity between studies was assessed with the Cochran Q and I^2^ statistics. For the qualitative interpretation of heterogeneity, I^2^ values of at least 50% were considered to represent substantial heterogeneity, while values of at least 75% indicated considerable heterogeneity, as per the Cochrane Handbook [14]. Publication bias (i.e. assessment of bias across studies) was graphically evaluated using a funnel plot, given that the Cochrane Handbook for Systematic Reviews of Interventions dictates as a rule of thumb that tests for funnel plot asymmetry should be used only when there are at least ten studies included in the meta-analysis [15].

We subsequently conducted subgroup analyses according (i) to the MRI protocol that was used for the measurement of brain volume changes as well as the assessment of brain atrophy and (ii) the year of the treatment on the studies that provided data on brain volume changes for both time-periods (0–12 months, 12–24 months). The mixed-effects model was used to calculate both the pooled point estimate in each subgroup and the overall estimates. According to the mixed-effects model, we used a random effects model (DerSimonian Laird) to combine studies within each subgroup and a fixed effect model (Mantel—Haenszel method) to combine subgroups and estimate the overall effect. We assumed the study-to-study variance (tau-squared) to be the same for all subgroups. Tau-squared was first computed within subgroups and then pooled across subgroups.

Finally, we performed post-hoc meta-regression analyses to evaluate both time alone (in placebo subgroups) and time-on-treatment (in treatment subgroups) as possible moderators of the percentage brain volume change. Univariate meta-regression analyses were performed using the random-effects model (Method of Moments). The regression lines derived from the aforementioned univariate meta-regression analyses were compared using the analysis of covariance (ANCOVA) to determine the effect of treatment on percentage brain volume change over time.

Statistical analyses were conducted using Review Manager (RevMan) Version 5.2 software (Copenhagen: The Nordic Cochrane Centre, The Cochrane Collaboration, 2012) and Comprehensive Meta-analysis Version 2 software (Borenstein M, Hedges L, Higgins J, Rothstein H, Biostat, Englewood NJ, 2005).

## Results

### Study selection and study characteristics

**Fig 1:**
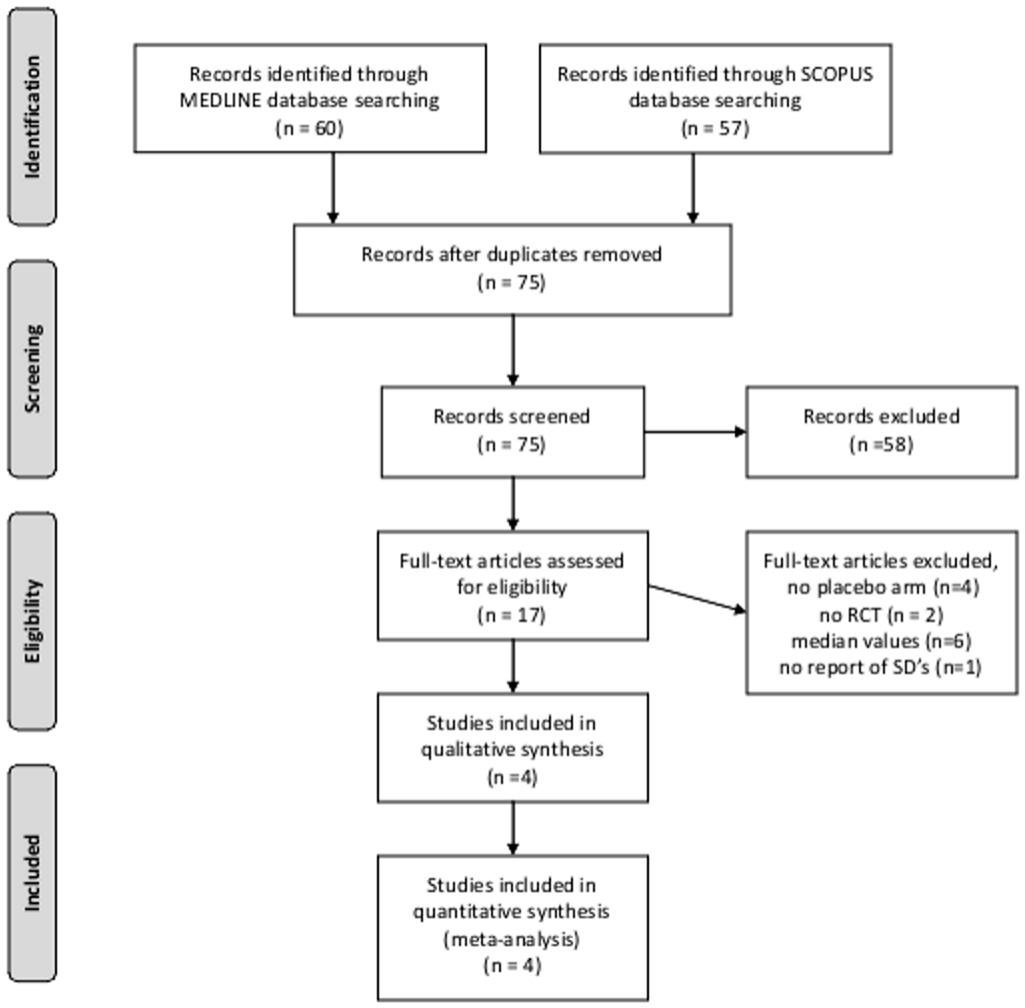
Flow chart presenting the selection of eligible studies.

Systematic search of MEDLINE and SCOPUS databases yielded 60 and 57 results respectively. Subsequent search in the CENTRAL Register of Controlled Trials retrieved no additional RCTs. After removing duplicates, the titles and abstracts from the remaining 75 studies were screened and 17 potentially eligible studies for the meta-analysis were retained. After retrieving the full-text version of the aforementioned 17 studies, 4 studies were excluded because they did not include a placebo subgroup and 2 studies because they were not RCT (cohort studies), 6 studies because they reported the brain volume changes in median values and 1 study because it did not report the SDs or other measures of dispersion (Table A in S1 File). In the final presentation of the literature search results, there was no conflict or disagreement between the 2 reviewers and the 4 studies that met the study protocol’s inclusion criteria (IMPROVE [7], FREEDOMS [8], E/C GASG [9], MSCRG [10]) were included both in the qualitative and quantitative synthesis (Fig. 1). The characteristics of the included studies, comprising 1819 patients (71% women, mean age: 36.5 years, mean baseline EDSS score: 2.4) are summarized in Table 1.

**Table 1:** Characteristics of the included in meta-analysis randomized clinical trials of patients with relapsing-remitting multiple sclerosis.

Author	Study name	Subgroup	Dose	No patients	Females	Age	bEDSS
De Stefano[7]	IMPROVE	INFb-1a (44 mcg)	3/ week (sc)	120	-	-	-
		Placebo	3/ week (sc)	60	-	-	-
Kappos[8]	FREEDOMS	Fingolimod (1.25mg)	1/ daily (pos)	429	68.8%	37.4±8.9	2.4±1.4
		Fingolimod (0.5mg)	1/ daily (pos)	425	69.6%	36.6±8.8	2.3±1.3
		Placebo	1/ daily (pos)	418	71.3%	37.2±8.6	2.5±1.3
Rovaris[9]	E/C GASG	GA (20mg)	1/ daily (sc)	113	77.0%	34.4±7.4	2.3±1.1
		Placebo	1/ daily (sc)	114	72.8%	34.0±7.6	2.4±1.2
Rudick[10]	MSCRG	INFb-1a (30 mcg)	1/ week (im)	68	76.5%	36.5± 7.2	2.32± 0.79
		Placebo	1/ week (im)	72	75%	36.4±7.1	2.38± 0.91

(sc: subcutaneously, pos: per os, im: intramuscularly, bEDSS: baseline Expanded Disability Status Scale)

Two of the studies [9,10] evaluating brain atrophy by means of the brain parenchymal fraction (BPF), a normalized measure of atrophy calculated as the ratio of brain parenchymal tissue volume to the total volume contained within the brain surface contour. More specifically, the neuroimaging protocol used an automated image analysis method that incorporates a three-dimensonal segmentation algorithm designed for brain surface detection and brain volume calculation [16]. The other two studies [12,13] used the Structural Image Evaluation using Normalization of Atrophy (SIENA) method, which provides an automated brain volume change analysis from baseline [17].

### Risk of bias for independent studies

Risk of bias in the included studies is summarized in Figs. A&B in S1 File. Overall, a low risk of bias was found within individual studies, except for the uncertainty of bias related to funding source according to Cochrane recommendations [18]. All study protocols were supported financially partly [10] or solely [7–9] by the pharmaceutical companies that produce and market the drug under consideration in each study. In one of them it was clearly stated in the methods section that the study data were collected by the investigators and analyzed by the sponsor pharmaceutical company [8], increasing thus the susceptibility to a possible bias. Allocation concealment was not clearly stated in the methods section of one study protocol [9], providing thus insufficient information to permit judgment.

### Overall analysis and subgroup analyses

**Fig 2:**
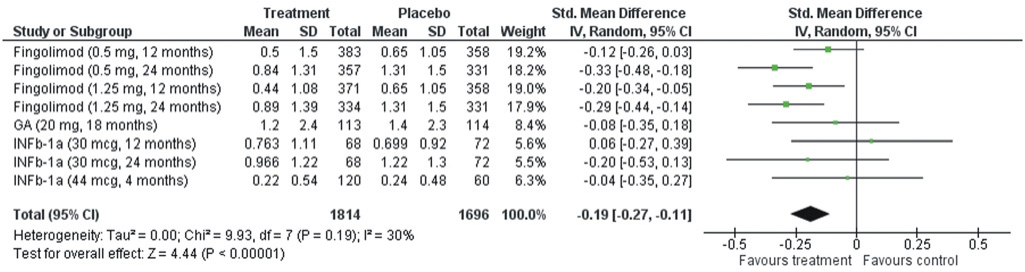
Percentage changes in mean brain volume in patients with relapsing-remitting multiple sclerosis receiving disease modifying therapy compared and those receiving placebo.

The mean percentage change in brain volume was found to be significantly lower in DMD treated patients versus placebo treated subgroup (SMD = -0.19, 95%CI: -0.27 –-0.11; p<0.001; Fig. 2). No evidence of heterogeneity was found between estimates (I^2^ = 30%, p = 0.19 by Cochran Q statistics). In the sentitivity analysis stratifying patients according to MRI protocol that was used for the measurement of the brain volume change, there was no evidence of heterogeneity in the estimates between the studies using the SIENA or the BPF method (Fig. C in S1 File).

**Fig 3:**
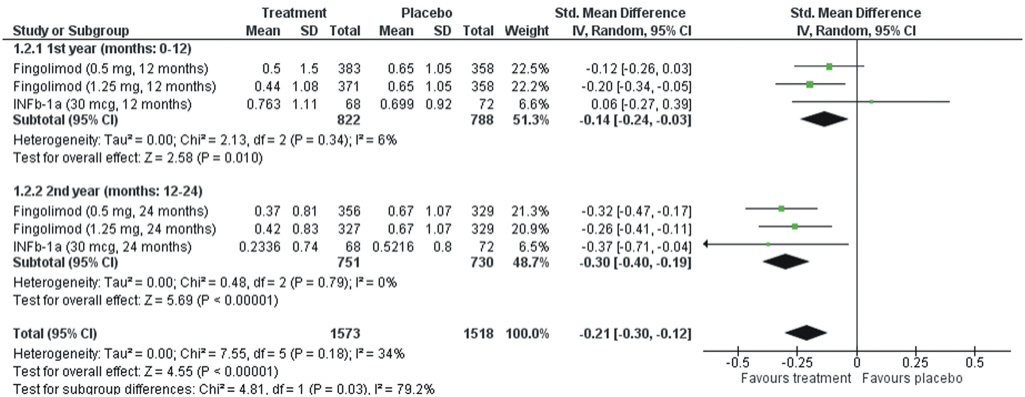
Subgroup analysis of the randomized clinical trials on percentage brain volume changes between the first and second year of the study.

In the subgroup analysis of the two studies that provided data on brain volume changes for both the first (0–12 months) and second (12–24 months) treatment year [8,10], the difference in percentage brain volume change between RRMS patients under treatment with Fingolimod (0.5 mg or 1.25 mg) or INFb-1a (30mcg) and RRMS patients randomized to placebo was greater (p = 0.03) in the second (SMD = -0.30; 95%CI: -0.40, -0.19) than in the first year of treatment (SMD = -0.14; 95%CI: -0.24, -0.03; Fig. 3), indicating that the beneficial effect of DMD on brain atrophy is enhanced during the second year of treatment.

Publication bias was evaluated using inspection of funnel plot asymmetry in view of the small number (n = 4) of included studies. The slight asymmetry observed on the funnel plot inspection can be attributed to the existing differences among studies with regard to neuroimaging protocols (Fig. D in S1 File) and treatment duration (Fig. E in S1 File), rather than in the presence of publication bias. To further investigate potential publication bias we also estimated Egger’s statistical test that did not reach the level of statistical significance (p = 0.09).

### Meta-regression analyses

**Fig 4:**
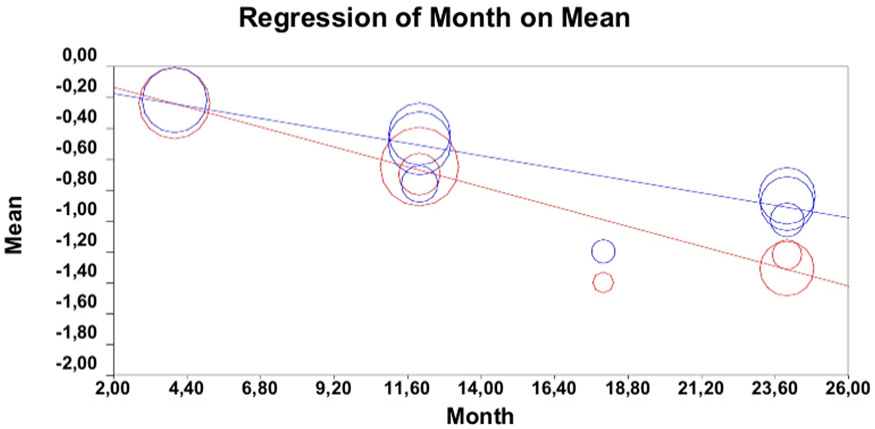
Meta-regression analyses of the percentage change in brain volume over time between treatment subgroups (blue lines) and placebo subgroups (red lines).

In meta-regression analyses, the percentage change in brain volume was found to be inversely associated with duration of observation period in both DMD (regression slope = -0.03; 95% CI: -0.04 –-0.02; p<0.001; Fig. 4, blue lines) and placebo subgroups (regression slope: -0.05; 95% CI: -0.06– -0.04; p<0.001; Fig. 4, red lines). By comparing the two meta-regression slopes, the rate of percentage brain volume loss over time was higher in placebo-treated patients than in patients receiving DMD (p = 0.017, ANCOVA).

## Discussion

The findings from the current systematic review and meta-analysis indicate that DMD appear to attenuate brain atrophy over time when compared with placebo. Moreover, their efficacy appears to be greater during the second year of treatment (in comparison to the first year) and linearly increases with longer treatment duration.

In untreated MS patients receiving placebo, the rate of atrophy is about 1%-1.5% of brain volume per year [19]. Given the fact that brain volume loss can be non-invasively and reproducibly detected and quantified by MRI [20], whole brain atrophy has recently emerged as an attractive measure of long-term tissue loss and as a substrate for clinical disability and therapy effectiveness [21,22]. In particular, brain volume loss has been correlated with disability progression and cognitive impairment in MS, the loss of grey matter volume being more closely correlated with clinical measures than loss of white matter volume [23]. However, for the time being brain volume loss may hardly be used as a decision making biological marker in the everyday clinical practice. Our analyses documented a modest beneficial effect of DMD on brain atrophy, with no evidence of heterogeneity across trials using different immunomodulatory treatments (sc INFb-1a, im INFb-1a, Fingolimod and Glatiramer Acetate) or different neuroimaging protocols (SIENA or BPF). Moreover, we documented low risk of selection, performance, detection, attrition and reporting biases using the validated, quality-control methodology of Cochrane collaboration for the assessment and quantification of biases in individual studies included in comprehensive meta-analyses [12]. Our findings add to the mounting literature endorsing extension of brain atrophy measurements beyond research studies to the routine management of MS patients and underscoring the need of their incorporation as secondary outcome measures in RCT [24,25].

Our sensitivity analyses indicated that the pooled beneficial effect of DMD on brain atrophy doubled during the second (SMD: -30) in comparison to the first year of treatment (SMD: -14). This observation may be attributed to an increase in non-tissue-related brain volume loss during the first 6–9 months of disease modifying therapies (termed pseudoatrophy), thought to be due to the resolution of inflammation and brain edema [19,26]. Interestingly, high-dose intravenous (IV) corticosteroids and especially natalizumab are associated with the highest decline in non-tissue-related brain volume loss due to their potent anti-inflammatory properties [19,26,27]. Thus, our observations suggest that the development of pseudoatrophy may confound MRI measurements of BV loss during the first year of treatment and an observation period of ≥ 2 years may be optimal to evaluate the potential beneficial effect of DMD on brain atrophy in future RCTs.

Our meta-regression analyses underline that the protective effect of DMD on brain volume loss is accentuated with longer treatment duration. This observation raises the clinical hypothesis that early initiation of DMD may extend the therapeutic time window leading to greater cumulative benefit in terms of brain atrophy prevention. This hypothesis is in agreement with pathology data indicating that acute axonal damage occurs early during disease course and consequently early treatment with disease-modifying therapies may prevent axonal and brain volume loss leading to disability progression [28]. Furthermore, RCT data also lends support to this hypothesis, since both sc INFb-1a [29] and glatiramer acetate [30] have been associated with attenuation of brain volume loss in comparison to placebo in patients with clinically isolated syndrome.

Certain limitations of this report need to be acknowledged. First, 8 study protocols were excluded from the quantitative analysis, as they reported the mean brain volume percentage changes in median values or did not provide the corresponding measures of dispersion in SDs. As a result the number of included studies was relatively small. Despite evaluating independent data from the different subgroups and/or time-points of each study in the overall analysis and meta-regression models, the findings of the present meta-analysis need to be reproduced in future using a larger number of RCTs. Second, even though quality control of the included studies suggests an overall low risk of bias, we cannot exclude bias related to funding source, as all study protocols had financial and/or other support from pharmaceutical industries with a clear conflict of interest on the study outcomes. Third, variable DMD (INFb-1a, GA, Fingolimod), in different dosages (Fingolimod 1.25mg/0.5 mg) and in different routes of administration (per os, intramuscularly, subcutaneously) were investigated in different RCTs. Thus, it is possible that the aforementioned differences in the treatment subgroups could be-at least partially- responsible for the identified correlations, even though no evidence of heterogeneity was detected among trials. Fourth, the imaging methods that were used for the measurement of brain volume change were not identical in the included studies and this may have confounded the reported associations. However, it should be kept in mind that no significant heterogeneity was detected between trials using different neuroimaging protocols in our sensitivity analyses. Finally, this meta-analysis evaluated only the effect of DMD on brain volume loss and our findings are not applicable to other treatment options currently evaluated in MS such as immunosuppressive or chemotherapy agents that have been shown to induce excessive and sustained brain volume reductions in MS patients [26,27,31].

## Supporting Information

S1 File MEDLINE search algorithm. **Table A**. List of the full-text excluded articles with the reasons for exclusion. **Supplemental references in S1 File. Fig. A.** Risk of bias summary: review authors’ judgments about each risk of bias item for each included study. **Fig. B.** Risk of bias graph: review authors’ judgments about each risk of bias item presented as percentages across all included studies. **Fig. C.** Subgroup analysis according to the imaging protocol that was used to measure the changes in brain volume. **Figs. D&E.** Funnel plots of the included studies.
(RTF)

## References

[1] Filippi M, Rocca MA (2010) MR imaging of gray matter involvement in multiple sclerosis: implications for understanding disease pathophysiology and monitoring treatment efficacy. AJNR Am J Neuroradiol 31:1171–1177. 10.3174/ajnr.A1944 20044503 PMC7965461

[2] Giorgio A, De Stefano N (2010) Cognition in multiple sclerosis: relevance of lesions, brain atrophy and proton MR spectroscopy. Neurol Sci 31:S245–8. 10.1007/s10072-010-0370-x 20635111

[3] Benedict RH, Carone DA, Bakshi R. Correlating brain atrophy with cognitive dysfunction, mood disturbances, and personality disorder in multiple sclerosis (2004) J Neuroimaging 14 (3 Suppl):36S–45S. 15228758 10.1177/1051228404266267

[4] Chard DT, Griffin CM, Parker GJ, Kapoor R, Thompson AJ, et al (2002) Brain atrophy in clinically early relapsing-remitting multiple sclerosis. Brain 125:327–337 11844733 10.1093/brain/awf025

[5] De Stefano N, Giorgio A, Battaglini M, Rovaris M, Sormani MP, et al (2010) Assessing brain atrophy rates in a large population of untreated multiple sclerosis subtypes. Neurology 74:1868–1876. 10.1212/WNL.0b013e3181e24136 20530323

[6] Sormani MP, Arnold DL, De Stefano N (2014) Treatment effect on brain atrophy correlates with treatment effect on disability in multiple sclerosis. Ann Neurol 75:43–9. 10.1002/ana.24018 24006277

[7] De Stefano N, Sormani MP, Stubinski B, Blevins G, Drulovic JS, et al (2012) Efficacy and safety of subcutaneous interferon β-1a in relapsing-remitting multiple sclerosis: further outcomes from the IMPROVE study. J Neurol Sci 312:97–101. 10.1016/j.jns.2011.08.013 21880336

[8] Kappos L, Radue EW, O’Connor P, Polman C, Hohlfeld R, et al (2010) A placebo-controlled trial of oral fingolimod in relapsing multiple sclerosis. N Engl J Med 362:387–401. 10.1056/NEJMoa0909494 20089952

[9] Rovaris M, Comi G, Rocca MA, Wolinsky JS, Filippi M, et al (2001) Short-term brain volume change in relapsing-remitting multiple sclerosis: effect of glatiramer acetate and implications. Brain 124:1803–12. 11522582 10.1093/brain/124.9.1803

[10] Rudick RA, Fisher E, Lee JC, Simon J, Jacobs L (1999) Use of the brain parenchymal fraction to measure whole brain atrophy in relapsing-remitting MS. Multiple Sclerosis Collaborative Research Group. Neurology 53:1698–704. 10563615 10.1212/wnl.53.8.1698

[11] Liberati A, Altman DG, Tetzlaff J, Mulrow C, Gotzsche PC, et al (2009) The prisma statement for reporting systematic reviews and meta-analyses of studies that evaluate health care interventions: Explanation and elaboration. J Clin Epidemiol 62:e1–34 10.1016/j.jclinepi.2009.06.006 19631507

[12] Higgins JP, Altman DG, Gotzsche PC, Jüni P, Moher D, et al (2011) The cochrane collaboration’s tool for assessing risk of bias in randomised trials. BMJ 343:d5928 10.1136/bmj.d5928 22008217 PMC3196245

[13] Cohen J (1988) Statistical Power Analysis for the Behavioral Sciences. Hillsdale, New Jersey: Lawrence Erlbaum Associates: Routledge.

[14] Deeks JJ, Higgins JP, Altman DG. Chapter 9: Analysing data and undertaking meta-analyses. Cochrane Handbook for Systematic Reviews of Interventions website.http://handbook.cochrane.org/chapter_9/9_analysing_data_and_undertaking_meta_analyses.htm. Updated March 2011. Accessed February 4th, 2014.

[15] Sterne JA, Sutton AJ, Ioannidis JP, Terrin N, Jones DR, et al (2011) Recommendations for examining and interpreting funnel plot asymmetry in meta-analyses of randomised controlled trials. BMJ 343:d4002 10.1136/bmj.d4002 21784880

[16] Fisher E, Cothren RM, Tkach JA, Masaryk TJ, Cornhill JF (1997) Knowledge-based 3D segmentation of MR images for quantitative MS lesion tracking. SPIE Med Imag 3034:599–610.

[17] Smith SM, Zhang Y, Jenkinson M, Chen J, Matthews PM, et al (2002) Accurate, robust, and automated longitudinal and cross-sectional brain change analysis. Neuroimage 17:479–89. 12482100 10.1006/nimg.2002.1040

[18] Bero LA (2013) Why the Cochrane risk of bias tool should include funding source as a standard item [editorial]. Cochrane Database of Systematic Reviews (12):ED000075 24575439 10.1002/14651858.ED000075 PMC10898502

[19] Zivadinov R, Stosic M, Cox JL, Ramasamy DP, Dwyer MG (2008) The place of conventional MRI and newly emerging MRI techniques in monitoring different aspects of treatment outcome. J Neurol 255 (Suppl 1):61–74 10.1007/s00415-008-1009-1 18317678

[20] Grassiot B, Desgranges B, Eustache F, Defer G (2009) Quantification and clinical relevance of brain atrophy in multiple sclerosis: a review. J Neurol 256:1397–412. 10.1007/s00415-009-5108-4 19353226

[21] Bermel RA, Bakshi R (2006) The measurement and clinical relevance of brain atrophy in multiple sclerosis. Lancet Neurol 5:158–170. 16426992 10.1016/S1474-4422(06)70349-0

[22] Barkhof F, Calabresi PA, Miller DH, Reingold SC (2009) Imaging outcomes for neuroprotection and repair in multiple sclerosis trials. Nat Rev Neurol 5:256–266. 10.1038/nrneurol.2009.41 19488083

[23] De Stefano N, Airas L, Grigoriadis N, Mattle HP, O’Riordan J, et al (2014) Clinical relevance of brain volume measures in multiple sclerosis. CNS Drugs 28:147–56. 10.1007/s40263-014-0140-z 24446248

[24] Arnold DL, De Stefano N (2013) Preventing brain atrophy should be the gold standard of effective therapy in multiple sclerosis (after the first year of treatment): Commentary. Mult Scler 19:1007–8. 10.1177/1352458513490550 23818020

[25] Rudick RA, Fisher E (2013) Preventing brain atrophy should be the gold standard of effective therapy in MS (after the first year of treatment): Yes. Mult Scler 19:1003–4. 10.1177/1352458513482385 23818018

[26] Zivadinov R, Reder AT, Filippi M, Minagar A, Stüve O, et al (2008) Mechanisms of action of disease-modifying agents and brain volume changes in multiple sclerosis. Neurology 71:136–44. 10.1212/01.wnl.0000316810.01120.05 18606968

[27] Khoury S, Bakshi R (2010) Cerebral pseudoatrophy or real atrophy after therapy in multiple sclerosis. Ann Neurol 68:778–9. 10.1002/ana.22254 21194148

[28] Kuhlmann T, Lingfeld G, Bitsch A, Schuchardt J, Brück W (2002) Acute axonal damage in multiple sclerosis is most extensive in early disease stages and decreases over time. Brain 125:2202–2212. 12244078 10.1093/brain/awf235

[29] Filippi M, Rovaris M, Inglese M, Barkhof F, De Stefano N, et al (2004) Interferon beta-1a for brain tissue loss in patients at presentation with syndromes suggestive of multiple sclerosis: a randomised, double-blind, placebo-controlled trial. Lancet 364:1489–1496. 15500893 10.1016/S0140-6736(04)17271-1

[30] Comi G, Martinelli V, Rodegher M, Moiola L, Leocani L, et al (2013) Effects of early treatment with glatiramer acetate in patients with clinically isolated syndrome. Mult Scler 19:1074–1083. 10.1177/1352458512469695 23234810

[31] Petzold A, Mondria T, Kuhle J, Rocca MA, Cornelissen J, et al (2010) Evidence for acute neurotoxicity after chemotherapy. Ann Neurol 68: 806–815. 10.1002/ana.22169 21194151

