# Supplementary material for: The Effect of Disease Modifying Therapies on Brain Atrophy in Patients with Relapsing-Remitting Multiple Sclerosis: A Systematic Review and Meta-Analysis
Source: PLoS One. 2015 Mar 10;10(3):e0116511. doi: 10.1371/journal.pone.0116511 (PMC4355592; doi:10.1371/journal.pone.0116511)
Supplement: S1 File — Table A. List of the full-text excluded articles with the reasons for exclusion. Supplemental references in S1 File. Fig. A. Risk of bias summary: review authors’ judgments about each risk of bias item for each included study. Fig. B. Risk of bias graph: review authors’ judgments about each risk of bias item presented as percentages across all included studies. Fig. C. Subgroup analysis according to the imaging protocol that was used to measure the changes in brain volume. Figs. D&E. Funnel plots of the included studies. (RTF) [file pone.0116511.s001.rtf]

Supporting Information S1 File

MEDLINE search algorithm
(("multiple sclerosis, relapsing-remitting"[MeSH Terms] OR ("multiple"[All Fields] AND "sclerosis"[All Fields] AND "relapsing-remitting"[All Fields]) OR "relapsing-remitting multiple sclerosis"[All Fields] OR ("relapsing"[All Fields] AND "remitting"[All Fields] AND "multiple"[All Fields] AND "sclerosis"[All Fields]) OR "relapsing remitting multiple sclerosis"[All Fields]) OR (("recurrence"[MeSH Terms] OR "recurrence"[All Fields] OR "relapsing"[All Fields]) AND ("multiple sclerosis"[MeSH Terms] OR ("multiple"[All Fields] AND "sclerosis"[All Fields]) OR "multiple sclerosis"[All Fields])) OR RRMS[All Fields]) AND ((("brain"[MeSH Terms] OR "brain"[All Fields]) AND ("atrophy"[MeSH Terms] OR "atrophy"[All Fields])) OR (("brain"[MeSH Terms] OR "brain"[All Fields]) AND volume[All Fields])) AND Randomized Controlled Trial[ptyp]


Tables
Table A. List of the full-text excluded articles with the reasons for exclusion

Excluded articles	Reasons for exclusion	
Calabrese et al1	Placebo subgroup not included	
Cohen et al2	Placebo subgroup not included	
Mikol et al3	Placebo subgroup not included	
Radue et al4	Placebo subgroup not included	
Fisher et al5	Cohort study (no randomization)	
Hardmeier et al6	Cohort study (no randomization)	
Filippi et al7	Brain volume in median values, not approved RRMS therapy	
Havrdova et al8	Brain volume in median values	
Horakova et al9	Brain volume in median values	
Leary et al10	Brain volume in median values	
Cohen et al11	Brain volume in median values	
Coles et al12	Brain volume in median values	
Miller et al13	No measures of dispersion reported	


Supplemental references
1. 	Calabrese M, Bernardi V, Atzori M, Mattisi I, Favaretto A, Rinaldi F, et al (2012) Effect of disease-modifying drugs on cortical lesions and atrophy in relapsing-remitting multiple sclerosis. Mult Scler 18:418-24. 
2. 	Cohen JA, Barkhof F, Comi G, Hartung HP, Khatri BO, Montalban X, et al (2010) Oral fingolimod or intramuscular interferon for relapsing multiple sclerosis. N Engl J Med 362:402-15.
3.	 Mikol DD, Barkhof F, Chang P, Coyle PK, Jeffery DR, Schwid SR, et al (2008) Comparison of subcutaneous interferon beta-1a with glatiramer acetate in patients with relapsing multiple sclerosis (the REbif vs Glatiramer Acetate in Relapsing MS Disease [REGARD] study): a multicentre, randomised, parallel, open-label trial.  Lancet Neurol 7:903-14. 
4. 	Radue EW, Stuart WH, Calabresi PA, Confavreux C, Galetta SL, Rudick RA, et al (2010) Natalizumab plus interferon beta-1a reduces lesion formation in relapsing multiple sclerosis. J Neurol Sci 292:28-35.
5. 	Fisher E, Rudick RA, Simon JH, Cutter G, Baier M, Lee JC, et al (2002) Eight-year follow-up study of brain atrophy in patients with MS. Neurology 59:1412-20.
6.	Hardmeier M, Wagenpfeil S, Freitag P, Fisher E, Rudick RA, Kooijmans M, et al (2005) Rate of brain atrophy in relapsing MS decreases during treatment with IFNbeta-1a.  Neurology 64:236-40.
7. 	Filippi M, Rocca MA, Pagani E, De Stefano N, Jeffery D, Kappos L, et al (2014) Placebo-controlled trial of oral laquinimod in multiple sclerosis: MRI evidence of an effect on brain tissue damage. J Neurol Neurosurg Psychiatry 85:851-8.
8. 	Havrdova E, Zivadinov R, Krasensky J, Dwyer MG, Novakova I, Dolezal  O, et al (2009) Randomized study of interferon beta-1a, low-dose azathioprine, and low-dose corticosteroids in multiple sclerosis. Mult Scler 15:965-76.
9.	 Horakova D, Dwyer MG, Havrdova E, Cox JL, Dolezal O, Bergsland N, et al (2009) Gray matter atrophy and disability progression in patients with early relapsing-remitting multiple sclerosis: a 5-year longitudinal study. J Neurol Sci 282:112-9. 
10.	Leary SM, Miller DH, Stevenson VL, Brex PA, Chard DT, Thompson AJ (2003) Interferon beta-1a in primary progressive MS: an exploratory, randomized, controlled trial. Neurology 60:44-51.
11.	Cohen JA, Coles AJ, Arnold DL, Confavreux C, Fox EJ, Hartung HP, et al (2012) Alemtuzumab versus interferon beta 1a as first-line treatment for patients with relapsing-remitting multiple sclerosis: a randomised controlled phase 3 trial. Lancet 380:1819-28.
12. 	Coles AJ, Twyman CL, Arnold DL, Cohen JA, Confavreux C, Fox EJ, et al (2012) Alemtuzumab for patients with relapsing multiple sclerosis after disease-modifying therapy: a randomised controlled phase 3 trial. Lancet 380:1829-39.
13. 	Miller DH, Soon D, Fernando KT, MacManus DG, Barker GJ, Yousry TA, et al (2007) MRI outcomes in a placebo-controlled trial of natalizumab in relapsing MS. Neurology 68:1390-401.


Figures

Figure A. Risk of bias summary: review authors' judgements about each risk of bias item for each included study.


Figure B. Risk of bias graph: review authors' judgements about each risk of bias item presented as percentages across all included studies 


Figure C. Subgroup analysis according to the imaging protocol that was used to measure the changes in brain volume 


Figures D&E. Funnel plots of the included studies
